# Caffeine Reduces 11β-Hydroxysteroid Dehydrogenase Type 2 Expression in Human Trophoblast Cells through the Adenosine A_2B_ Receptor

**DOI:** 10.1371/journal.pone.0038082

**Published:** 2012-06-11

**Authors:** Saina Sharmin, Haiyan Guan, Andrew Scott Williams, Kaiping Yang

**Affiliations:** Children’s Health Research Institute and Lawson Health Research Institute, Departments of Obstetrics, Gynaecology, Physiology and Pharmacology, The University of Western Ontario, London, Ontario, Canada; Ottawa Hospital Research Institute and University of Ottawa, Canada

## Abstract

Maternal caffeine consumption is associated with reduced fetal growth, but the underlying molecular mechanisms are unknown. Since there is evidence that decreased placental 11β-hydroxysteroid dehydrogenase type 2 (11β-HSD2) is linked to fetal growth restriction, we hypothesized that caffeine may inhibit fetal growth partly through down regulating placental 11β-HSD2. As a first step in examining this hypothesis, we studied the effects of caffeine on placental 11β-HSD2 activity and expression using our established primary human trophoblast cells as an *in vitro* model system. Given that maternal serum concentrations of paraxanthine (the primary metabolite of caffeine) were greater in women who gave birth to small-for-gestational age infants than to appropriately grown infants, we also studied the effects of paraxanthine. Our main findings were: ***(1)*** both caffeine and paraxanthine decreased placental 11β-HSD2 activity, protein and mRNA in a concentration-dependent manner; ***(2)*** this inhibitory effect was mediated by the adenosine A_2B_ receptor, since siRNA-mediated knockdown of this receptor prevented caffeine- and paraxanthine-induced inhibition of placental 11β-HSD2; and ***(3)*** forskolin (an activator of adenyl cyclase and a known stimulator of 11β-HSD2) abrogated the inhibitory effects of both caffeine and paraxanthine, which provides evidence for a functional link between exposure to caffeine and paraxanthine, decreased intracellular levels of cAMP and reduced placental 11β-HSD2. Taken together, these findings reveal that placental 11β-HSD2 is a novel molecular target through which caffeine may adversely affect fetal growth. They also uncover a previously unappreciated role for the adenosine A_2B_ receptor signaling in regulating placental 11β-HSD2, and consequently fetal development.

## Introduction

Fetal growth restriction (FGR) occurs in 5–10% of all pregnancies, and it is a leading cause of perinatal morbidity and mortality [1]–[4]. Moreover, there is accumulating evidence that FGR infants are at a greater risk of developing cardiovascular and metabolic diseases, cognitive defects, and certain cancers later in life [5]–[8]. Although it is known that a number of fetal, maternal and environmental factors contribute to FGR, the etiology of FGR remains largely unknown (i.e., approx. 60% of FGR are classified as idiopathic) [1], [9], [10]. A number of epidemiological studies indicate that maternal caffeine consumption is associated with an increased risk of giving birth to small-for-gestational age babies, although such an association is not robust [11]–[15]. Indeed, there is a dose-dependent association between mean caffeine consumption of >200 mg/day over the course of pregnancy and reductions in birth weight [12], [16], [17]. Furthermore, elevated maternal serum concentrations of paraxanthine, the primary metabolite of caffeine, were associated with reduced fetal growth [18], [19]. Taken together, these findings suggest that both caffeine and/or its main metabolite may adversely affect fetal growth and development, but the underlying molecular mechanisms are unclear.

Given that caffeine is arguably one of the most commonly consumed substances during pregnancy [20], [21], the study of the molecular mechanisms by which caffeine and its main metabolite paraxanthine adversely affect fetal growth is a key component of our overall efforts at unraveling the factors and their underlying molecular mechanisms that contribute to impaired fetal growth and development. Pharmacokinetic studies have demonstrated that caffeine and its metabolites pass readily through the placenta to the fetus [22], [23]. It is also known that maternal caffeine consumption during pregnancy is associated with reduced intervillous placental blood flow [24] and impaired placental development [25], suggesting that the placenta is an important target by which caffeine influences fetal growth and development. However, the precise molecular targets within the placenta upon which caffeine acts are unclear.

Knowing that reduced 11β-hydroxysteroid dehydrogenase type 2 (11β-HSD2, encoded by the *HSD11B2* gene) is linked to FGR in humans [26]–[28] and *HSD11B2* null mice exhibit FGR phenotype [29], coupled with the findings that the biological function of caffeine is largely mediated by adenosine receptors whose activation leads to changes in cAMP levels [30], which are known to alter placental 11β-HSD2 [31], [32], we hypothesized that caffeine might reduce fetal growth in part by down-regulating placental 11β-HSD2. As a first step in testing this hypothesis, we studied the effects of caffeine on placental 11β-HSD2 using our established primary human trophoblast cells as an *in vitro* model system. Given the association between higher maternal serum concentrations of paraxanthine and reduced fetal growth [18], [19], we also examined the effects of paraxanthine. Finally, we investigated the molecular mechanisms underlying the effects of caffeine and paraxanthine on placental 11β-HSD2.

## Materials and Methods

### Reagents

[1,2,6,7−^3^H(N)]-Cortisol (80 Ci/mmol) was purchased from DuPont Canada Inc. (Markam, ON). Non-radioactive cortisol and cortisone were obtained from Steraloids Inc. (Wilton, NH). Caffeine, paraxanthine (also known as 1,7-dimethylxanthine) and forskolin were purchased from Sigma-Aldrich Canada Ltd. (Oakville, ON). Polyester-backed thin-layer chromatography (TLC) plates were obtained from Fisher Scientific Ltd. (Nepean, ON). All solvents used were from VWR Canlab (Mississauga, ON). Cell culture supplies were obtained from either Invitrogen Life Technologies (Burlington, ON) or Fisher Scientific. General molecular biology reagents were from Invitrogen or Pharmacia Canada Inc. (Baie D’Urte, QC).

### Primary Trophoblast Cell Cultures

Placental trophoblast cells were isolated using a modification of the method of Kliman [33], as described [34]. Ethics approval for the entire study was obtained from the University of Western Ontario Ethics Board for Health Sciences Research Involving Human Subjects, and informed written consent was obtained from all participants involved in this study. Briefly, human placentas were obtained from uncomplicated pregnancies at term after elective cesarean section. Villous tissues were dissected free from fetal membranes and blood vessels, rinsed in 0.9% w/v NaCl, and digested with 0.125% v/v trypsin and 0.02% w/v deoxyribonuclease-I (Sigma) in DMEM containing 0.05% w/v streptomycin and gentamicin (Invitrogen) for 60–80 min. The placental cells were loaded onto a 5–70% v/v Percoll (Sigma) gradient at step increments of 5% Percoll, and centrifuged at 2,500 *g* for 20 min to separate different cell types. Cytotrophoblasts between the density markers of 1.049 and 1.062 g/ml were collected and plated in 24-well plates at a density of 1.35×10^6^ cells/ml in M199 containing 10% FCS (Invitrogen). The cells were maintained at 37°C in humidified 5% CO_2_-95% air (20% O_2_) for 48 hours. We have shown previously that the isolated cytotrophoblasts will differentiate into syncytiotrophoblasts over 48 h of culture under conditions of the present study [34]. After the end of 48 h, the trophoblast cells (in triplicate) were treated for 48 h (or as indicated otherwise) with various compounds in the medium containing 2% FCS. Controls, also in triplicate, received equivalent volume of vehicle (ethanol or DMSO). Under conditions of the present study, cell viability was not affected by either caffeine or paraxanthine treatment.

### Assay of 11β-HSD2 Activity - Radiometric Conversion Assay

The level of 11β-HSD2 activity in intact cells following different treatment regimes was determined by measuring the rate of cortisol to cortisone conversion, as described previously [34]. Briefly, human trophoblast cells were incubated for 1 h at 37°C in serum-free medium containing approx. 100,000 dpm [^3^H]-cortisol and 100 nM unlabelled cortisol. At the end of incubation, the medium was collected, and steroids extracted. The extracts were dried, and the residues resuspended. A fraction of the resuspension was spotted on a TLC plate that was developed in chloroform/methanol (9∶1, v/v). The bands containing the labelled cortisol and cortisone were identified by UV light of the cold carriers, cut out into scintillation vials and counted in Scintisafe™ Econo 1 (Fisher Scientific). The rate of cortisol to cortisone conversion was calculated, and the blank values (defined as the amount of conversion in the absence of cells) were subtracted, and expressed as percentage of control. Results are shown as mean ± SEM.

### Determination of 11β-HSD2 Protein - Western Blot Analysis

Levels of 11β-HSD2 protein were determined with standard western blot analysis, as described previously [35]. Briefly, human trophoblast cells were lysed in SDS Sample Buffer (62.5 mM Tris-HCl, pH 6.8, 2% w/v SDS, 10% v/v glycerol, 50 mM DTT, and 0.01% w/v bromophenol blue). Equal concentrations of the whole cell lysates were subjected to a standard 12% SDS-PAGE. After electrophoresis, proteins were transferred to PVDF transfer membrane (Amersham Hybond™-P, Cat# RPN303F) using a Bio-Rad Mini Transfer Apparatus. Non-specific antibody binding was blocked with 5% w/v milk in TTBS (0.1% v/v Tween-20 in TBS) for 1 h at room temperature. Membranes were then hybridized with primary antibody (rabbit anti-human 11β-HSD2 antibody, a generous gift from Dr. Z. Krozowski [36], 0.25 µg/ml; and rabbit anti-human GAPDH antibody, Cell Signaling, Cat #2118, 1∶5000) overnight at 4°C. After 3×5 min washes with TTBS, the membrane was incubated with the appropriate HRP-labelled second antibody (R & D Systems, Cat# HAF008, 1∶1000) for 1 h at room temperature. Proteins were detected by chemiluminescence (Western Lightning™ Plus-ECL, PerkinElmer Life and Analytical Sciences, Cat# NEL103001). Densitometry was performed on the radiographs, and the level of 11β-HSD2 protein was expressed as percent of controls.

### Assessment of 11β-HSD2 mRNA – Real-time Quantitative RT-PCR

The relative abundance of 11β-HSD2 mRNA was assessed by a two-step real-time quantitative RT-PCR (qRT-PCR), as described previously [37].

Briefly, total RNA was extracted from cultured cells using RNeasy Mini Kit (QIAGEN Inc., Mississauga, ON) coupled with on-column DNase digestion with the RNase-Free DNase Set (QIAGEN) according to the manufacturer’s instructions. One microgram of total RNA was reverse-transcribed in a total volume of 20 µl using the High Capacity complimentary deoxyribonucleic acid (cDNA) Archive Kit (Applied Biosystems, Forest City, CA) following the manufacturer’s instructions. For every RT reaction set, one RNA sample was set up without reverse-transcriptase enzyme to provide a negative control. Gene transcript levels of 28S rRNA (house-keeping gene) and 11β-HSD2 were quantified separately by custom-designed SYBR Green I chemistry-based assays [38]. Briefly, primers (300 nM each) for human 11β-HSD2 (sense: 5′-GGC CAA GGT TTC CCA GTG A-3′, antisense: 5′-CAG GGT GTT TGG GCT CAT GA-3′) and those (100 nM) for 28S rRNA (sense: 5′-TTG AAA ATC CGG GGG AGA G-3′, antisense: 5′-ACA TTG TTC CAA CAT GCC AG-3′) were designed with the Primer Express Software (Applied Biosystems), and the optimal concentrations were determined empirically. The SYBR Green I assays were performed with the SYBR Green PCR Master Mix (Applied Biosystems) and a modified universal thermal cycling condition (2 min at 50°C and 10 min at 95°C, followed by 40 cycles of 10 sec each at 95°C, 60°C and 72°C) with the standard disassociation/melting parameters (15 sec each at 95°C, 60°C and 95°C) on the ABI PRISM 7900HT^®^ Sequence Detection System (Applied Biosystems). The specificity of the SYBR Green I assays was verified by performing melting curve analyses and by subsequent sequencing of the PCR products.

Levels of 11β-HSD2 mRNA and 28S rRNA in each RNA sample were quantified by the relative standard curve method (Applied Biosystems). Briefly, standard curves for 11β-HSD2 and 28S rRNA were generated by performing a dilution series of the untreated control cDNA. For each RNA sample, the relative amount of 11β-HSD2 mRNA and 28S rRNA was obtained, and the ratio of 11β-HSD2 mRNA to 28S rRNA was calculated. For each experiment, the amount of 11β-HSD2 mRNA under various treatment conditions is expressed relative to the amount of transcript present in the untreated control.

### Analysis of Adenosine Receptor Expression - RT–PCR

Expression of the four human adenosine receptors, ADORA_1_, ADORA_2A_, ADORA_2B_ and ADORA_3_ was analyzed by standard RT-PCR [39]. Briefly, total RNA was isolated from cultured trophoblast cells as well as term placental tissues using RNeasy Mini Kit (Qiagen, Mississauga, ON, Canada) coupled with on-column DNase digestion with the RNase-Free DNase Set (Qiagen) according to the manufacturer’s instructions. One microgram of total RNA was reverse transcribed in a volume of 20 µl with the High Capacity cDNA Archive Kit (Applied Biosystems, Foster City, CA, USA), following the manufacturer’s instructions. For every RT reaction, one RNA sample was set-up without reverse transcriptase enzyme to provide a negative control against possible genomic DNA contamination. The primers specific for human ADORA_1_, ADORA_2A_, ADORA_2B_ and ADORA_3_ as well as their expected product sizes are shown in Table 1. PCR reactions were performed for 35 cycles with denaturing at 95°C, annealing at 55°C, and extension at 72°C. PCR products were confirmed with standard restriction enzyme digestions and sequencing analysis.

**Table 1:** PCR primers for the four human adenosine receptors and human GAPDH.

Gene name	Primer sequence	Product size (bp)
*ADORA_1_*	Forward: 5′- TCTGGGCGGTGAAGGTGAAC Reverse: 5′- AGTTGCCGTGCGTGAGGAAG	750
*ADORA_2A_*	Forward: 5′- TGCTTCGTCCTGGTCCTCAC Reverse: 5′- GCTCTCCGTCACTGCCAT	754
*ADORA_2B_*	Forward: 5′- CCCTTTGCCATCACCATCAG Reverse: 5′- CCTGACCATTCCCACTCTTGA	781
*ADORA_3_*	Forward: 5′- GCGCCATCTATCTTGACATCTTTT Reverse: 5′- CTTGGCCCAGGCATACAGG	460
*GAPDH*	Forward: 5′- ACCACAGTCCATGCCATCAC Reverse: 5′- TCCACCACCCTGTTGCTGTA	450

### siRNA-mediated Knockdown of ADORA_2A_ and ADORA_2B_ Expression

To determine if ADORA_2A_ and/or ADORA_2B_ is involved in mediating the effects of caffeine and paraxanthine on placental 11β-HSD2, a siRNA-mediated knockdown approach was utilized [35], [39]. Briefly, the isolated human trophoblast cells were plated on 24-well plates and cultured under standard conditions for 48 h. Cells were then transfected with 100 nM of siRNA targeting human ADORA_2A_ (5′-CCA UGU GUU AAA GGA AUA UTT, 3′-TTG GUA CAC AAU UUC CUU AUA; GenePharma, Shanghai, China) and ADORA_2B_ (5′-GGC CAU UCU UCU GUC ACA UTT, 3′-TTC CGG UAA GAA GAC AGU GUA; GenePharma) in Opti-MEM I medium (Invitrogen) containing 2 µl/well of Lipofectamine™ 2000 (Invitrogen), following the manufacturer’s instructions. Cells were also transfected in an identical manner with the transfection agent alone to serve as controls. At 48 h post-transfection, cells were collected for RT-PCR, as described above. Alternatively, 12 h after transfection, cells were treated for 48 h with 500 µM of caffeine or paraxanthine. At the end of treatment, levels of 11β-HSD2 protein were determined by western blot analysis.

### Statistical Analyses

Results are presented as mean ± SEM of four to six independent experiments (i.e., tissues from different patients), as indicated. Data were analyzed using one-way ANOVA followed by Tukey’s post-hoc test, or Student’s *t* test as indicated. Significance was set at p<0.05. Calculations were performed using SPSS software version 9.0 (Chicago, IL).

## Results

### Effects of Caffeine on 11β-HSD2 Activity and Expression

**Figure 1:**
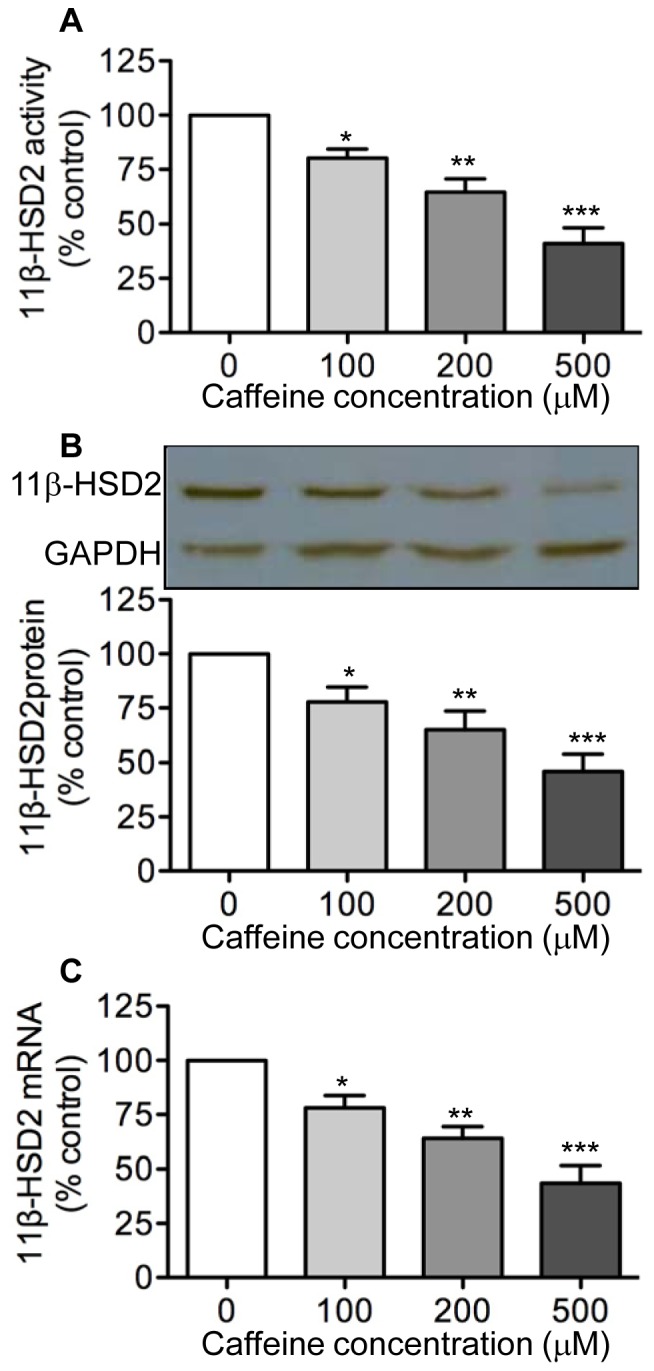
Concentration-dependent effects of caffeine on 11β-HSD2 activity and expression. Human trophoblast cells were treated with increasing concentration of caffeine (100–500 µM) for 48 h. At the end of treatment, levels of 11β-HSD2 activity in intact cells (**A**) as well as levels of 11β-HSD2 protein (**B**) and 11β-HSD2 mRNA (**C**) were determined by a standard radiometric conversion assay, western blotting, and qRT-PCR, respectively. Data are presented as mean ± SEM of four to five independent experiments (*P<0.05, **P<0.01, ***P<0.001 vs. control).

Given that changes in enzyme activity are more biologically meaningful than alterations in enzyme expression, we first examined the effects of caffeine on placental 11β-HSD2 activity by treating isolated human trophoblast cells with increasing concentrations of caffeine (100–500 µM) for 48 h. As shown in **Fig. 1A**, this treatment resulted in a concentration-dependent decrease in levels of 11β-HSD2 activity such that a significant decrease (p<0.05) was seen at 100 µM with a progressive decrease at higher caffeine concentrations. We then determined if this inhibitory effect of caffeine was mediated by altered 11β-HSD2 expression. We found a similar concentration-dependent decrease in levels of both 11β-HSD2 protein and mRNA following caffeine treatment (**Fig. 1B and 1C**).

### Effects of Paraxanthine on 11β-HSD2 Activity and Expression

**Figure 2:**
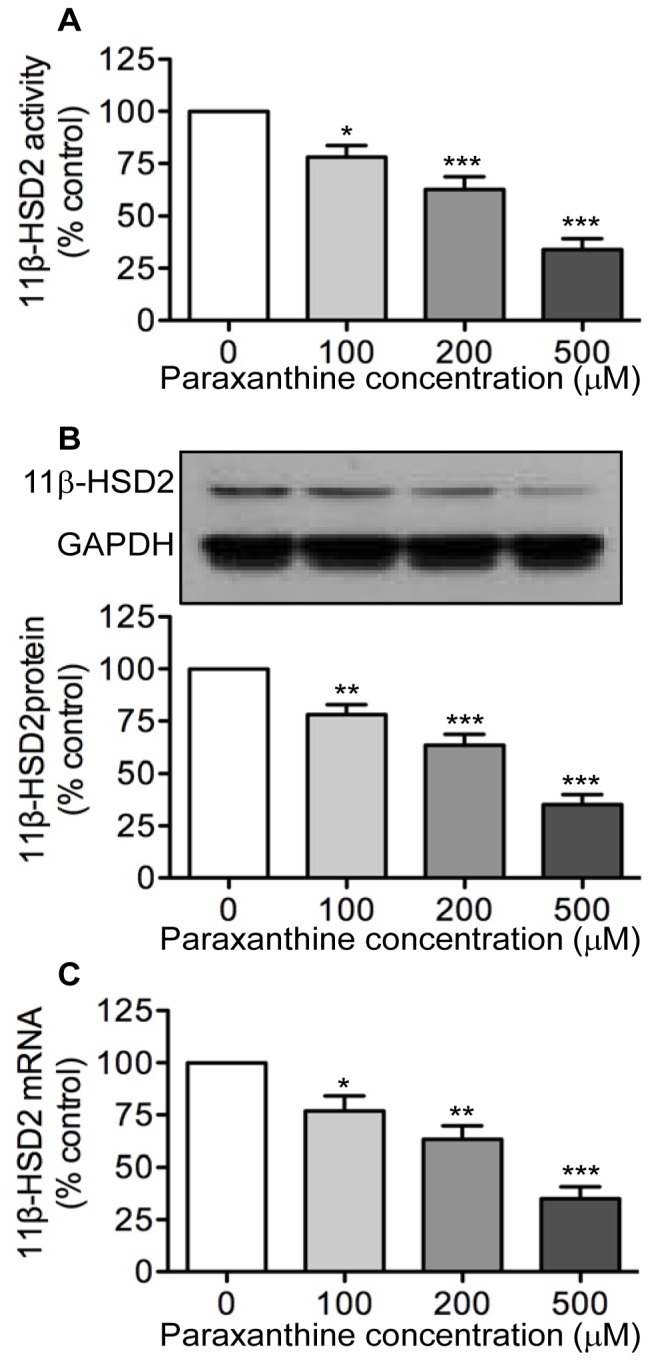
Concentration-dependent effects of paraxanthine on 11β-HSD2 activity and expression. Human trophoblast cells were treated with increasing concentration of paraxanthine (PX; 100–500 µM) for 48 h. At the end of treatment, levels of 11β-HSD2 activity in intact cells (**A**) as well as levels of 11β-HSD2 protein (**B**) and 11β-HSD2 mRNA (**C**) were determined by a standard radiometric conversion assay, western blotting, and qRT-PCR, respectively. Data are presented as mean ± SEM of four to five independent experiments (*P<0.05, **P<0.01, ***P<0.001 vs. control).

Given that paraxanthine is the primary metabolite of caffeine in humans [40], coupled with the findings that maternal serum concentrations of paraxanthine were higher in women who gave birth to FGR infants than to appropriately grown infants [18], [19], we determined whether this metabolite also exerts an inhibitory effect on placenta 11β-HSD2. Indeed, treatment of trophoblast cells with increasing concentrations of paraxanthine (100–500 µM) led to a similar concentration-dependent decrease in levels of 11β-HSD2 activity (**Fig. 2A**), protein (**Fig. 2B**) and mRNA (**Fig. 2C**).

### Expression of Adenosine Receptors in Human Trophoblast Cells

**Figure 3:**
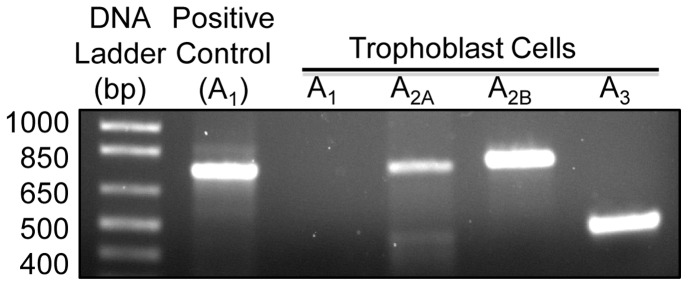
Expression of adenosine receptor mRNA in cultured human trophoblast cells. Total RNA was extracted from cultured trophoblast cells and from placental villi at term (to serve as a positive control for ADORA_1_). One microgram of RNA was used in a standard RT-PCR to amplify mRNA for the four human adenosine receptors, ADORA_1_, ADORA_2A_, ADORA_2B_ and ADORA_3_. A fraction of the RT-PCR products was subjected to electrophoresis on a 1.2% agarose gel. This figure shows the results of one representative experiment.

As a first step in defining the receptors that mediate the inhibitory effects of caffeine and paraxanthine on placental 11β-HSD2, we examined the expression of the four-adenosine receptors (ADORA_1_, ADORA_2A_, ADORA_2B_ and ADORA_3_) in cultured human trophoblast cells, because there is robust evidence for their involvement in mediating a wide range of caffeine functions [20]. As shown in **Fig. 3**, the mRNAs encoding ADORA_2A_, ADORA_2B_ and ADORA_3_ were readily detectable in cultured human trophoblast cells. In contrast, the ADORA_1_ mRNA was undetectable in trophoblast cells, although it was readily detected in total RNA extracts from human placental tissues (served as positive control) (**Fig. 3**).

### Effects of siRNA-mediated Knockdown of ADORA_2B_ and ADORA_2A_ Expression on Caffeine and Paraxanthine Inhibition of 11β-HSD2

**Figure 4:**
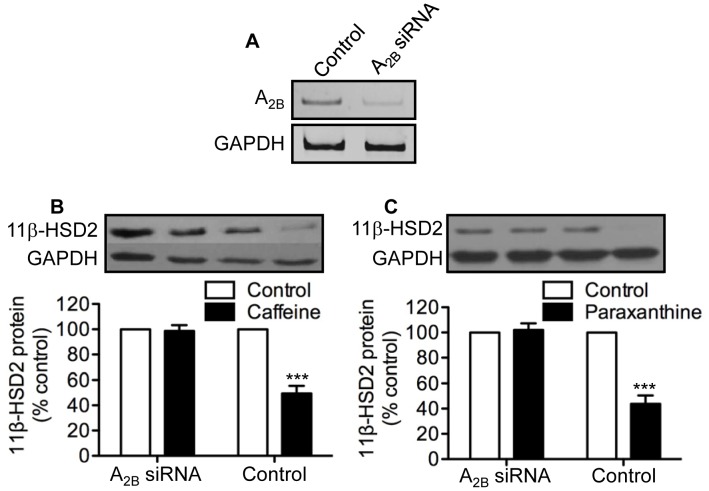
Effects of siRNA-mediated knockdown of ADORA_2B_ on caffeine and paraxanthine inhibition of 11β-HSD2 expression. Human trophoblast cells were transfected with 100 nM of ADORA_2B_ siRNA or the transfection agent alone to serve as control. Forty-eight hours after transfection, cells were lysed, and total RNA extracted and subjected to a semi-quantitative RT-PCR to determine levels of ADORA_2B_ mRNA (**A**). GAPDH was used as a control to show the specificity of siRNA mediated knockdown of ADORA_2B_. Alternatively, 12 h after transfection, cells were treated for 48 h with or without 500 µM of caffeine (**B**) or paraxanthine (**C**). At the end of treatment, levels of 11β-HSD2 11β-HSD2 protein were determined by western blot analysis. Data are presented as mean ± SEM of four to five independent experiments (***P<0.001 vs. control).

**Figure 5:**
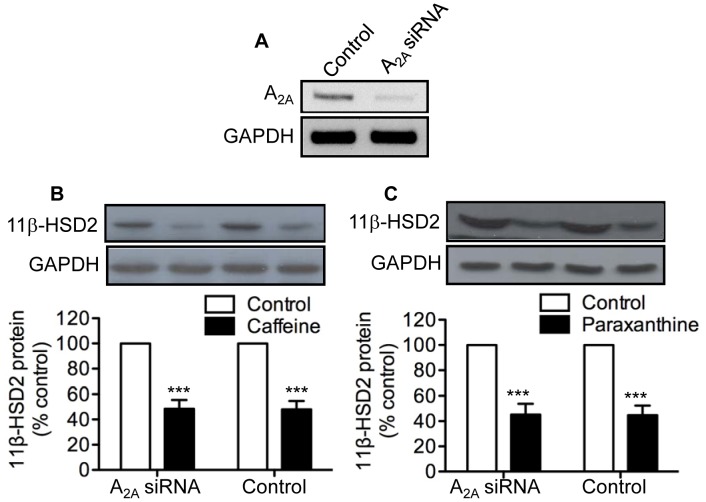
Effects of siRNA-mediated knockdown of ADORA_2A_ on caffeine and paraxanthine inhibition of 11β-HSD2 expression. Human trophoblast cells were transfected with 100 nM of ADORA_2A_ siRNA or the transfection agent alone to serve as control. Forty-eight hours after transfection, cells were lysed, and total RNA extracted and subjected to a semi-quantitative RT-PCR to determine levels of ADORA_2A_ mRNA (**A**). GAPDH was used as a control to show the specificity of siRNA mediated knockdown of ADORA_2A_. Alternatively, 12 h after transfection, cells were treated for 48 h with or without 500 µM of caffeine (**B**) or paraxanthine (**C**). At the end of treatment, levels of 11β-HSD2 11β-HSD2 protein were determined by western blot analysis. Data are presented as mean ± SEM of four independent experiments (***P<0.001 vs. control).

Knowing that caffeine functions as an antagonist for ADORA_1_, ADORA_2A_ and ADORA_2B_ but not ADORA_3_ [20], coupled with the lack of a detectable level of ADORA_1_ mRNA in cultured human trophoblast cells (**Fig. 3**), we reasoned that ADORA_2A_ and/or ADORA_2B_ might mediate the inhibitory effects of caffeine and paraxanthine on placental 11β-HSD2. To examine this possibility, we used a loss of gene function approach. As shown in **Fig. 4A**, transfection of trophoblast cells with a siRNA specific for human ADORA_2B_ resulted in an 80% reduction in ADORA_2B_ mRNA abundance. Having established the efficacy of siRNA-mediated knockdown of ADORA_2B_ expression, we then determined the effects of caffeine and paraxanthine on 11β-HSD2 protein in the ADORA_2B_ knockdown trophoblast cells. As shown in **Fig. 4B and 4C**, transient transfection of human trophoblast cells with ADORA_2B_-specific siRNA resulted in the complete loss of the ability of both caffeine and paraxanthine to down-regulate 11β-HSD2 protein expression. In contrast, both caffeine and paraxanthine were equally effective in reducing 11β-HSD2 protein levels in trophoblast cells transfected with and without ADORA_2A_-specific siRNA (**Fig. 5**).

### Effects of Forskolin on Caffeine and Paraxanthine Inhibition of 11β-HSD2

**Figure 6:**
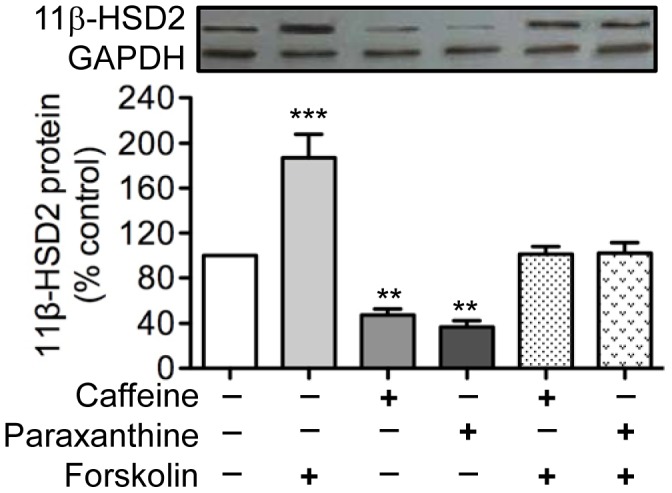
Effects of forskolin on caffeine and paraxanthine inhibition of 11β-HSD2 expression. Human trophoblast cells were pretreated for 2 h with 20 µM of forskolin, and were then treated with 500 µM of caffeine or paraxanthine for 48 h. At the end of treatment, levels of 11β-HSD2 protein were determined by western blot analysis. Data are presented as mean ± SEM of three independent experiments (**P<0.01, ***P<0.001 vs. control).

Given that the activation of ADORA_2B_ leads to increases in intracellular levels of cAMP [30], which are known to up-regulate placental 11β-HSD2 expression [31], [32], we determined the effects of forskolin, an activator of adenyl cyclase, on the inhibitory effects of caffeine and paraxanthine. We reasoned that if caffeine signals through ADORA_2B_ in trophoblast cells, caffeine treatment should result in decreases in cAMP levels and the addition of forskolin should counteract this effect of caffeine, and consequently block caffeine-induced inhibition of placental 11β-HSD2. Indeed, treatment of trophoblast cells with forskolin completely abrogated the inhibitory effect of caffeine on 11β-HSD2 expression (**Fig. 6**). Similarly, forskolin also prevented paraxanthine-induced decreases in levels of 11β-HSD2 protein (**Fig. 6**).

## Discussion

The present study demonstrates for the first time that caffeine and its main metabolite, paraxanthine, reduce 11β-HSD2 activity and expression in cultured human trophoblast cells. It also reveals that the inhibitory effects of caffeine and paraxanthine on placental 11β-HSD2 are mediated by the adenosine A_2B_ receptor. Given that both maternal caffeine consumption and reduced placental 11β-HSD2 are linked to impaired fetal growth, the present findings suggest that placental 11β-HSD2 may be a novel molecular target through which caffeine may adversely affect fetal growth and development.

Although several previous experimental studies reported that maternal caffeine administration during pregnancy led to reduced fetal and placental weight in rats [41], mice [42], and monkeys [43], which corroborates and extends the human epidemiological observations [15], the molecular mechanisms by which caffeine inhibits fetal growth had never been explored. Therefore, in the present study we tested the hypothesis that caffeine reduces fetal growth partly through inhibition of placental 11β-HSD2. Using our established primary human trophoblast cells as an *in vitro* model system, we showed that both caffeine and its primary metabolite paraxanthine decreased 11β-HSD2 activity and expression. Importantly, concentrations of caffeine and paraxanthine used in the present study are comparable to, albeit 2–3 times (at the minimal effective concentration –100 µM) higher than, those reported in human blood circulation. For instance, a mean blood caffeine concentration of ∼ 22 µM was reported in pregnant women at 36 weeks of gestation [44], while a plasma concentration of 40 µM caffeine and 20 µM paraxanthine was observed in humans after ingestion of 250 mg of caffeine [45]. Recently, one study showed that plasma levels of caffeine were between 1–50 µM in umbilical cords of preterm newborns [46]. Thus, if these *in vitro* findings could be extrapolated to human pregnancies *in vivo*, they would suggest that caffeine might inhibit fetal growth at least in part by down-regulating placental 11β-HSD2 directly and/or indirectly through its major metabolite, paraxanthine. Obviously, future studies will be required to determine if these *in vitro* findings can be confirmed in an animal model *in vivo*. Furthermore, the effects of caffeine on cell fate (e.g., proliferation, syncytialization, apoptosis of trophoblast cells) remain to be explored.

At micromolar concentrations, both caffeine and its principal metabolite paraxanthine function as adenosine receptor antagonists [20]. Four distinct adenosine receptors have been characterized, and they are designated as ADORA_1_, ADORA_2A_, ADORA_2B_, and ADORA_3_ [30]. Previous studies showed that all four adenosine receptors (both mRNA and protein) were detectable in human placentas, and their levels of expression were elevated in preeclamptic placentas [47]. Furthermore, one study showed that ADORA_2B_ immunoreactivity was present in syncytiotrophoblast cells of human placenta [48]. In addition, ADORA_3_ regulated matrix metalloproteinase 2 expression in preeclamptic placental explant cultures [49]. As a first step in determining if adenosine receptors were involved in mediating the inhibitory effects of caffeine and paraxanthine on placental 11β-HSD2, we examined the expression of the four-adenosine receptors in cultured human trophoblast cells with standard RT-PCR. We showed that although ADORA_1_ mRNA was undetectable, mRNAs encoding ADORA_2A_, ADORA_2B_, and ADORA_3_ were readily detected in trophoblast cells. The lack of detectable mRNA encoding ADORA_1_ in cultured trophoblast cells cannot be attributed to any technical issues, because it was readily detected in our positive control (i.e., RNA extracted from whole placental tissues), but seems to contradict with one previous study reporting the presence of ADORA_1_ protein in human placental trophoblast cells [49]. In the absence of control data showing the specificity of ADORA_1_ antibody used in the previous study, it is difficult to reconcile this discrepancy. Obviously, the resolution of this discrepancy awaits future independent studies.

Given that ADORA_1_ mRNA was undetectable in trophoblast cells, coupled with the fact that caffeine does not signal through ADORA_3_ [20], we postulated that the inhibitory effects of caffeine and paraxanthine were likely mediated by ADORA_2A_ and/or ADORA_2B_. To test this hypothesis, we used a loss of gene function approach in which the expression of ADORA_2A_ and ADORA_2B_ was inhibited with siRNA-mediated knockdown. We showed that both caffeine and paraxanthine completely lost their ability to reduce 11β-HSD2 expression in trophoblast cells transfected with a siRNA specific for ADORA_2B_. In marked contrast, caffeine and paraxanthine were equally effective in suppressing 11β-HSD2 expression in trophoblast cells transfected with and without a siRNA specific for ADORA_2A_, suggesting that the effects of caffeine and paraxanthine on placental 11β-HSD2 were mediated by ADORA_2B_.

Having established the involvement of ADORA_2B_ in mediating the inhibitory effects of caffeine and paraxanthine on placental 11β-HSD2, we then explored the role of cAMP in ADORA_2B_ signaling. Although there is robust evidence that activation of ADORA_2B_ leads to increases in cAMP levels [30], which are known to stimulate 11β-HSD2 expression [31], [32], and caffeine functions as an ADORA_2B_ antagonist [20], there is no direct evidence linking caffeine (or paraxanthine) exposure to decreases in cAMP levels and reduced 11β-HSD2. To determine if such a link exists, we treated trophoblast cells with caffeine or paraxanthine in the presence and absence of forskolin, an activator of adenyl cyclase and a known stimulator of 11β-HSD2 [31], [32]. We showed that forskolin abrogated the inhibitory effects of both caffeine and paraxanthine on placental 11β-HSD2. Taken together, our present findings provide evidence that caffeine and paraxanthine, via antagonizing ADORA_2B_, decrease intracellular cAMP levels and inhibit 11β-HSD2 expression and activity in cultured human trophoblast cells.

In summary, the present study identifies placental 11β-HSD2 as a molecular target through which caffeine may reduce fetal growth. It also uncovers a novel role for the adenosine A_2B_ receptor signaling in regulating placental 11β-HSD2, and consequently fetal development.
